# Critical roles of conventional dendritic cells in autoimmune hepatitis via autophagy regulation

**DOI:** 10.1038/s41419-019-2217-6

**Published:** 2020-01-13

**Authors:** Xiaoli Fan, Ruoting Men, Chen Huang, Mengyi Shen, Tingting Wang, Yasmeen Ghnewa, Yun Ma, Tinghong Ye, Li Yang

**Affiliations:** 1 Department of Gastroenterology and Hepatology, Sichuan University-Oxford University Huaxi Gastrointestinal Cancer Centre, West China Hospital, Sichuan University, Chengdu, China; 2 Institute of Liver Studies, King’s College London Faculty of Life Sciences and Medicine at King’s College Hospital, London, UK; 3 Laboratory of Liver Surgery, State Key Laboratory of Biotherapy/Collaborative Innovation Center for Biotherapy, West China Hospital, Sichuan University, Chengdu, China

**Keywords:** Phosphorylation, Autoimmune diseases

## Abstract

Autoimmune hepatitis (AIH) is a necroinflammatory disease associated with interactive cell populations of the innate and adaptive immune systems. The contribution of conventional dendritic cells (cDCs) to AIH and the underlying mechanism remain poorly understood. The frequency of peripheral mature cDCs increased in AIH patients and was positively correlated with disease severity. In experimental autoimmune hepatitis (EAH), hepatic accumulation of mature cDCs was observed, along with an increase in the periphery. Sequentially, bone marrow-derived dendritic cells (BMDC) from EAH mice exhibit more proinflammatory function than those from control mice. In vitro, ConA treatment promotes the maturation of BMDCs, which are characterized by higher expression of MHC-II, costimulatory molecules and cytokine secretion. ConA also induced the expression of autophagy-related protein and the formation of autophagosomes in DCs. To further investigate whether ConA-induced DC activation is associated with autophagy, we utilized 3-MA and bafilomycin A1 to block autophagy flux and accessed the maturation and function of DCs induced by ConA. 3-MA and bafilomycin A1 inhibited the mature status and proinflammatory cytokine secretion and diminished the proliferation and differentiation of CD4+ T cells when ConA-induced BMDCs cocultured CD4+ T cells. We demonstrated that cDCs contribute to the pathogenesis of AIH through excessive maturation. Aberrant autophagy flux plays a vital role in the immunogenic maturation of cDCs in AIH, and tolerogenic cDCs by inhibition of autophagy flux can be exploited as a new therapeutic approach for AIH.

## Introduction

Autoimmune hepatitis (AIH) is a chronic necroinflammatory disease of the liver that is characterized by histological interface hepatitis, hypergammaglobulinemia and the production of autoantibodies and could rapidly lead to cirrhosis and end-stage-liver disease if left untreated^1^. During AIH, self-tolerance (also termed homeostatic processes) is impaired, resulting in Kupffer cell (KC)-, neutrophil-, monocyte-, and T-cell-mediated inflammatory and immune reactions, which are implicated in the pathogenesis of autoimmune liver injury^2,3^. However, the vital role of conventional dendritic cells (DCs) in the initiation and extension of AIH is not fully understood.

As critical regulators of innate immunity, DCs are professional antigen presenting cells displaying the unique capability to activate naive T cells and play important roles in the immune response^4^. DCs are heterogeneous, differing in origin, location, function and migratory pathways^5^. Infections or inflammatory stimuli can also affect their function and generation. Conventional DCs (cDCs) are a DC subsets with a dendritic form that exhibit DC functions in a steady state. cDCs account for 1% of hepatic nonparenchymal cells (NPC). DCs from a healthy liver exhibit a decreased ability to capture antigens and stimulate T cells; they are also reported to be less immunogenic than their splenic counterparts. A series of studies have found that hepatic DCs play a regulatory role in liver disease. Using CD11c–DTR Tg mice, Bamboat et al. eported that during liver ischemia/reperfusion (I/R) injury, cDC production of IL-10 could suppress inflammatory monocyte function and then reduce liver injury^6^. Additionally, cDC depletion in DTR mice in acetaminophen (APAP) hepatotoxicity could exacerbate liver injury in a manner independent of neutrophils, natural killer (NK) cells or inflammatory mediators^7^. For other liver disorders, the dysfunction of DCs has also been described in previous studies^8^. However, the characterization of cDCs during AIH and the underlying mechanism remain to be elucidated.

In this study, we dissected mature cDC subsets in the peripheral blood of AIH patients and in the peripheral blood and liver of experimental autoimmune hepatitis (EAH) mice to investigate the precise engagement of mature cDC subsets in the pathogenesis of AIH. We also observed that the maturation and function of cDCs profoundly contribute to AIH via an autophagy-dependent mechanism, which can be ameliorated by blocking the autophagy flux. The current study may stimulate subsequent studies investigating the precise role of cDCs in the pathogenesis and progression of AIH.

## Materials and methods

### Patients

A total of 29 peripheral blood samples were obtained from patients with AIH between Jan. 2016 to Mar. 2018 at the Division of Gastroenterology & Hepatology, West China Hospital, Sichuan University (Sichuan, China). All patients met the diagnostic criteria for AIH (a score ≥10 indicates probable AIH before treatment, and a score ≥12 indicates probable AIH before treatment) issued by the International Autoimmune Hepatitis Group (1999)^9,10^. Their clinical characteristics are listed in Table 1. Twenty-one healthy subjects were studied as controls. Plasma was also retained for analysis of cytokine profiles. The study was reviewed and approved by the Ethics Committee of the West China Hospital, Sichuan University (no. 2013221). All enrolled patients have gave their written informed consent.

**Table. 1:** Clinical characteristics of the AIH patients recruited.

Clinical characteristics	Amount
Age, years	52.3 ± 10.4
Female	27/29 (93.1%)
Liver function indexes	
TBil, umol/L	91.2 ± 110.7
ALT, IU/L	237.9 ± 200.9
AST, IU/L	286.5 ± 194.0
Immunoglobulin	
IGG, IU/L	29.7 ± 9.5
ANA (+, N%)	29 (100%)

### Animals

Female C57BL/6 mice (aged 8–10 weeks; 19–22 g) were obtained from the Sichuan University Laboratory Animal Center (Chengdu, China). The mice were housed in a specific-pathogen-free (SPF) facility with a consistent room temperature and humidity and had free access to standard laboratory chow and water one week before the experiment. All animal experiments were approved by the Institutional Animal Care and Treatment Committee of Sichuan University in China.

### Main reagents

ConA and lipopolysaccharide (LPS) were purchased from Sigma–Aldrich (St. Louis, MO, USA). Antibodies against CD11C (Cat #97585) and Beclin-1 (Cat #3495) were purchased from Cell Signaling Technology (Danvers, MA, USA). LC3B (sc-376404), ATG7 (sc-376212) and HLA-DR (53319) were purchased from Santa Cruz Biotechnology (Europe), and β-actin was purchased from ZSGB-BIO (Beijing, China). MHC-II (abs 125192) was purchased from Absin (Shanghai, China). P62 (R1309-8) was purchased from HUABIO (Hangzhou, China). ELISA kits were from MultiSciences (Hangzhou, China). Anti-CD4 microbeads and fluorochrome-coupled antibodies for CD11C, CD4, CD3, HLA-DR, CD69, MHC-II, CD80 and CD86 were from Biolegend (San Diego, CA, USA). Mouse recombinant GM-CSF, IL-4, tumor necrosis factor-α (TNF-α), and IL-33 were obtained from Novoprotein (China). Additionally, 5-(and-6)-carboxyfluorescein diacetate succinimidyl ester (CFSE) was purchased from Invitrogen (San Diego). Mouse lymphocyte separation medium (7211011) was obtained from DAKEWE (Shenzhen, China). 3-MA was purchased from Selleck (Houston, TX, USA). Bafilomycin A1 was from MedChemExpress (NJ, USA).

### Experimental design

The EAH model was established at 12 h after an injection of ConA with a dose of 20 mg/kg through the tail vein, which was based on our previous study and other reported articles^11–14^. To evaluate the EAH model, the mice were divided into two groups: (1) Mice were given a single intravenous injection of normal saline as a vehicle control. (2) Mice were given a single intravenous injection of ConA at a dose of 20 mg/kg body weight and were sacrificed at 12 h after ConA administration. The distribution of each group strictly followed the principle of randomization. In the animal study, *n* = 5 for each group. Researchers who were blinded to the experimental groups and protocol evaluated the results.

### Flow cytometry analysis

Venous blood was collected aseptically from patients and healthy volunteers. Red blood cells in the human peripheral blood were lysed and washed twice with phosphate-buffered saline (PBS). In addition, single-cell suspensions of blood and liver of mice were obtained 12 h after ConA administration by using mechanical and enzymatic dispersion as described previously^15^. In general, the peripheral blood and liver were harvested. Red blood cells in the peripheral blood were lysed. A single-cell suspension of the liver was mechanically disrupted and then enzymatically digested with 1 mg/mL collagenase IV. Then, 1 × 10^6^ freshly prepared cells were suspended in 100 μl of PBS and stained with different combinations of fluorochrome-coupled antibodies. Data were acquired by flow cytometry on a FACS Canton II (BD Biosciences) or NovoCyte (ACEA) and analyzed by FlowJo software.

### Generation of bone marrow dendritic cells

C57BL/6 mouse bone marrow-derived DCs (BMDCs) were generated as previously described^16^. Briefly, bone marrow cells were harvested from the femur and tibia of 8- to 12-wk-old mice. Cells were cultured in RPMI 1640 medium containing l-glutamine, sodium pyruvate, nonessential amino acids, and 50 nM 2-mercaptoethanol, supplemented with 10% FCS, 10 ng/mL GM-CSF, and 10 ng/mL IL-4. The medium was half-refreshed every 2–3 d. After 8 days of culture, the nonadherent and loosely adherent cells were harvested as immature BMDCs (imDCs). The purity of imDCs was ~80% (Supplementary Fig. 1A). Then, the imDCs were cultured for an additional day with ConA, LPS, or without stimuli as a control. Subsequently, imDCs were also pretreated with 5 mM of 3-MA or 100 nM of bafilomycin, 0.5 h or 1 h, respectively, prior to harvest after ConA stimulation. The resulting DCs were thoroughly washed and used for phenotypical (flow cytometry) and functional characterization (co-culture assays), and supernatants were frozen for cytokine evaluation by ELISA analysis.

### Mixed lymphocyte reaction

CD4+ T cells from the spleens of C57 mice were isolated using mouse lymphocyte separation medium and then purified with magnetic-activated cell-sorting beads (Biolegend, San Diego, CA). The purity of CD4+ cells was ~95% (Supplementary Fig. 1B). On day 8, immature BMDCs were incubated with ConA for 24 h, washed to clear residual stimulant, and then treated with mitomycin (25 μg /mL, Solarbio) for 30 min (37 °C). BMDCs from different groups were cocultured for 72 h with purified CD4+ T cells at a 1:10 ratio in the presence of 50 μg/mL ovalbumin (OVA) protein in complete RPMI 1640 culture medium containing 10% FBS supplemented with sodium pyruvate, l-glutamine, and nonessential amino acids. For the proliferation assay, the T cells were pre-labeled with CFSE (37 °C) for 15 min and then cultured with the BMDCs for 72 h. Data were acquired by flow cytometry.

### Liver function and cytokine assay

Retro-orbital blood samples were collected from the mice. The plasma was separated by centrifugation at 2000 rpm for 10 min. Alanine aminotransferase (ALT) and aspartate transaminase (AST) levels were measured by an automatic dry biochemical analyzer (Hitachi Auto Analyzer 7170, Japan). The levels of IL-12 and IFN-γ in the murine plasma were analyzed by ELISA kits according to the manufacturer’s instructions.

### Histopathology assay and immunohistochemistry

Liver samples were fixed in 4% buffered paraformaldehyde for 48 h. Sections (3 μm) on slides were deparaffinized with xylene, rehydrated with decreasing concentrations of ethanol, and then the 3 μm sections were stained with hematoxylin and eosin (H&E). For immunohistochemical analysis, sections were deparaffinized and rehydrated, followed by antigen retrieval. Then, the sections were incubated in 3% bovine serum albumin (BSA) for 30 min and primary antibody CD11C, CD4, MHC-II or HLA-DR overnight at 4 °C. After extensive washing with PBS, the sections were incubated with secondary antibodies for 50 min at room temperature. Finally, the liver sections were observed by light microscopy, and the slides were evaluated by at least two professional researchers in a double-blinded assessment.

### Transmission electron microscopy

Fragments from the BMDCs with or without ConA treatment were fixed in 2.5% glutaraldehyde, fixed in 1% osmium acid, dehydrated in an alcohol gradient, embedded in epoxy resin, and sliced by a microtome. The sections were stained, and images were taken by transmission electron microscopy (TEM) (HT7700, Hitachi, Japan).

### Western blot analysis

Whole cell extractions were obtained using RIPA lysis buffer in the presence of protease inhibitors and phosphatase inhibitor for 30 min on ice, followed by centrifugation at 13,300 rpm at 4 °C for 15 min to clear the lysates, and then the supernatant of the lysate was harvested. Protein concentration was determined by the Bradford Protein Assay Kit, using known amounts of BSA to standardize protein concentration and equalize before loading. Equivalent amounts of total protein (usually 30~40 μg) were separated on an SDS-PAGE gel and then transferred to polyvinylidene difluoride (PVDF) membranes (Amersham Bioscience, Piscataway, N.J.). After blocking, the membrane was incubated with primary antibodies overnight at 4 °C. The blots were incubated with an HRP-conjugated secondary antibody at 37 °C for 1 h. Finally, the membranes were washed twice and detected using an enhanced chemiluminescence system.

### mRFP-GFP-tagged LC3

Cells were transfected with a fluorescent tandem-tagged GFP-mRFP-LC3 plasmid (Addgene, 21074) according to the manufacturer’s instructions. DC2.4 cell line were detached by gentle pipetting, washed twice in OptiMEM medium (Thermo Fischer Scientific, Waltham, MA), and seeded in 24-well plates (5 × 10^4^ cells/well) in OptiMEM medium. Cells were transfected for 24 h, using INVI DNA RNA Transfection Reagent (Invigentech) according to the manufacturer’s instructions. The cells then were detected after ConA treating for 8 h. GFP and mRFP expression was visualized with a confocal microscope (Zeiss Microsystems, Germany). Autophagic flux was detected via analyzing the punctate pattern of GFP and mRFP.

### Monodansylcadaverine (MDC) staining

Autophagic vacuoles formed in the cells were detected by MDC staining. DC2.4 cell line were seeded at a density of 5 × 10^4^ cells/well on cover slips in 24-well plates and incubated overnight to allow adherence. Then, the cells were treated with saline or ConA for 8 h. The cells were then washed three times with PBS and incubated with MDC (50 μM) for 30 min in the dark at 37 °C. Next, excess MDC was washed away, and the cells on the cover slips were washed with PBS and fixed with 4% paraformaldehyde for 15 min. Autophagic vacuoles formed in the cells were analyzed by fluorescence microscopy, according to the guidelines of the manufacturer.

### Immunofluorescence staining

DC2.4 was stimulated by ConA for 8 h. Later, the cells were harvested and stained with anti-LC3-II mAbs and anti-P62 mAbs for overnight, followed by fixed in 4% paraformaldehyde for 20 min, per-meabilized with 0.3% Triton- X-100 for 15 min, and blocked in 5% BSA with 0.1% Tween-20. Subsequently, the cells were incubated with a corresponding fluorescence-labeled secondary antibody for 1.5 h and were viewed on a microscope (Zeiss Microsystems, Germany).

### Statistics

Descriptive and analytical statistics were performed using the SPSS (version 22.0) software package. Statistical significance between groups was determined by two-tailed Student’s *t*-test. The data are presented as the mean ± SD. Significance was accepted at *p* < 0.05.

## Results

### Circulating mature cDCs were increased in AIH patients and positively correlated with AIH severity

**Fig. 1:**
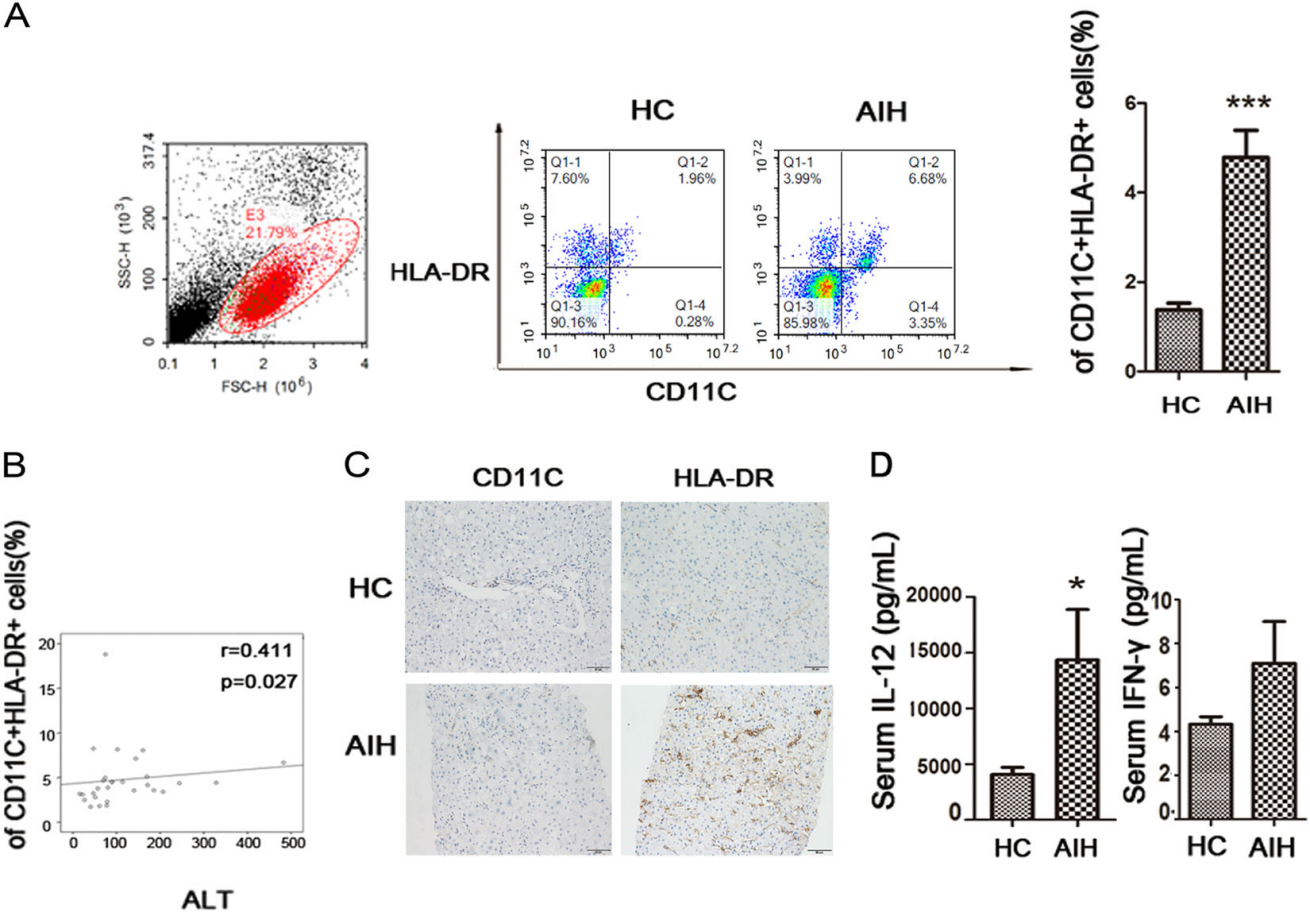
Circulating mature cDCs was increased in AIH patients. **a** Analysis of CD11C + HLA-DR + DCs in the peripheral blood of HCs and AIH patients. **b** Frequency of circulating mature cDCs is positively correlated with hepatic inflammation marker in AIH. **c** Representative immunohistochemistry staining of CD11C and HLA-DR in the livers of HC and AIH patients. **d** ELISA analysis of cytokines in the peripheral blood of HCs and AIH patients. Original magnification: x200. Bars show the mean ± SD; *p < 0.05 and ***p < 0.001.

Twenty-one healthy subjects and twenty-nine AIH patients were studied. The clinical characteristics of these subjects are listed in Table 1. First, we compared circulating CD11C+HLA-DR+DCs and CD3+CD4+ activated T cells between AIH and healthy controls (HCs). The frequency of mature DCs and activated T cells in peripheral lymphocytes significantly increased in AIH patients compared to the healthy controls. The levels of mature cDCs in the AIH group increased by ~3.5-fold compared with those in the HC group (*p* < 0.001) (Fig. 1a), while the frequency of CD3 + CD4 + activated T cells was also elevated in AIH (Supplementary Fig. 2). The correlation between the frequency of mature cDCs and markers of liver inflammation was analysed. We found that there was a positive correlation of peripheral mature cDCs frequency with ALT (Fig. 1b), while there was no correlation between peripheral mature cDC frequency and total bilirubin, AST and IgG values (data not shown). As CD11C and HLA-DR are markers for cDCs, we assessed intrahepatic cDCs by immunohistochemical method. We noted significant accumulation of CD11C + and HLA-DR + cells in liver of AIH patient (Fig. 1c). To validate that the mature cDC-related cytokines were involved, we performed cytokine analysis for human IL-12 and IFN-γ. We compared the serum levels of cytokines between AIH patients and the HC group to determine the important role of mature cDCs in AIH development. A significant increase in circulating IL-12 was observed in untreated AIH patients when compared with the HC group (*p* < 0.05), while no significant difference in serum IFN-γ was observed between untreated AIH patients and the HC cohort (Fig. 1d). Overall, mature cDCs were accumulated peripheral and liver in AIH and produced pro-inflammatory cytokines.

### Circulating and intrahepatic mature cDC accumulation in EAH

Con A-induced hepatitis is a well-established T-cell-mediated murine model that mimics human AIH, and immune cells are activated after ConA injection, accompanied by the release of many cytokines and activated lymphocyte infiltration to aggravate liver injury^17,18^. Given the increased cDC infiltration in human AIH, we performed further analysis in an AIH mouse model to illuminate the underlying mechanism.

**Fig. 2:**
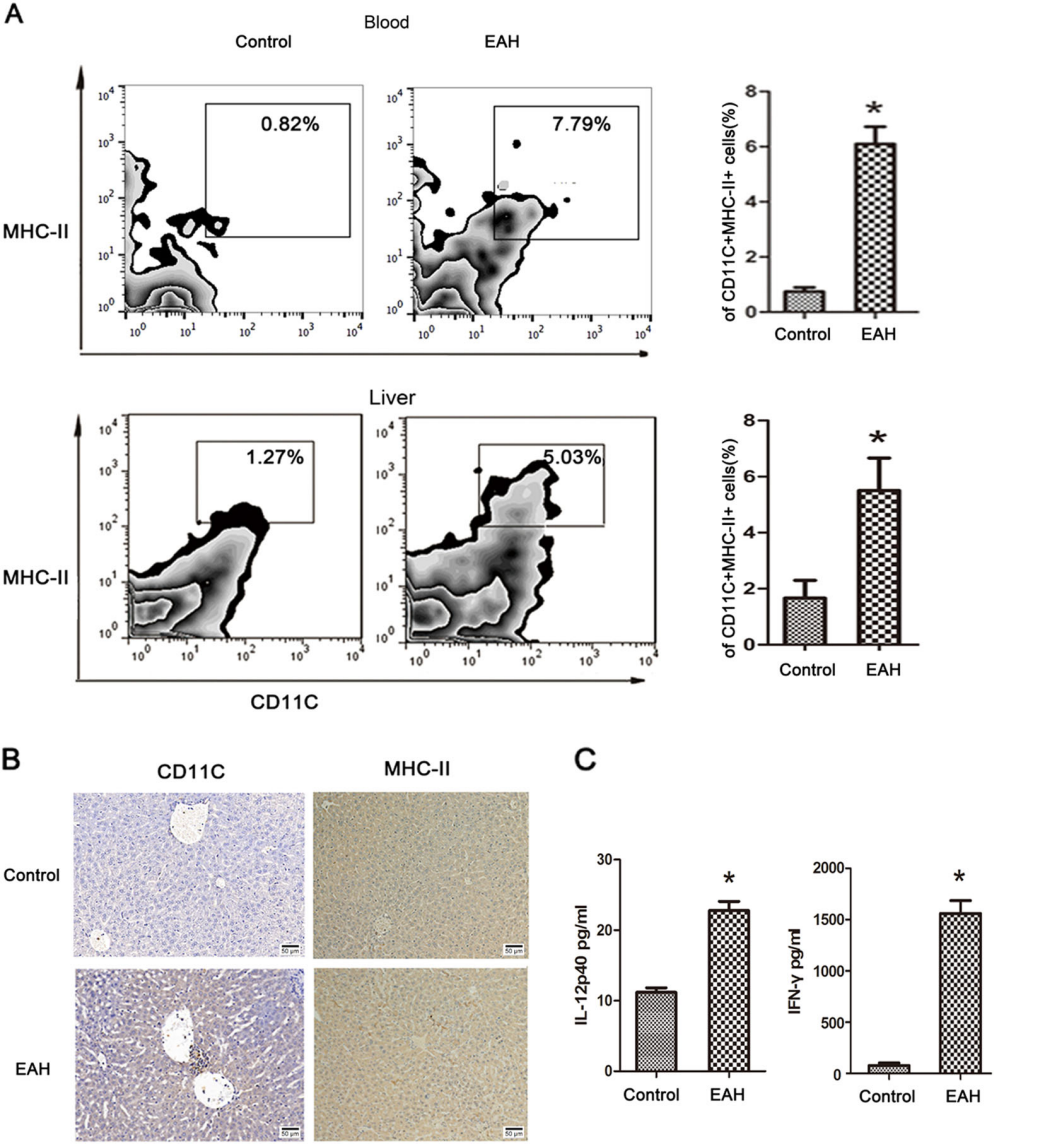
Circulating and intrahepatic cDCs accumulation in EAH model. **a** Frequency of mature cDCs in blood and liver tissues in EAH, respectively; **b** IHC staining of CD11C and MHC-II in the hepatic tissues between the two groups. Original magnification: x200; **c** Serum IL-12 and IFN-γ. Bars show the mean ± SD; *p < 0.05 versus control.

To investigate the role of cDCs in the pathogenesis of EAH, we developed a mouse model of ConA-induced AIH as in our previous study^11,14,19^. As shown by liver function and H&E, EAH was successfully established after ConA injection in EAH model (Supplementary Fig. 3A-C). The frequency of mature cDCs (CD11C+ MHC-II+) in blood and liver were determined by flow cytometry. We found that the frequency of mature cDCs in the blood and liver was significantly increased during the active phase of EAH model when compared to normal control (Fig. 2a), along with the frequency of activated T cells (Supplementary Fig. 3D–F). Furthermore, we confirmed that CD11C+ and MHC-II+ cells infiltrated the portal areas (Fig. 2b). Also, the levels of IL-12 and IFN-γ were up-regulated in the peripheral blood of EH model (Fig. 2c). These observations confirmed that cDCs were recruited in peripheral blood and liver in the immune microenvironment of EAH model.

### Pro-inflammatory function of BMDCs from EAH mice

**Fig. 3:**
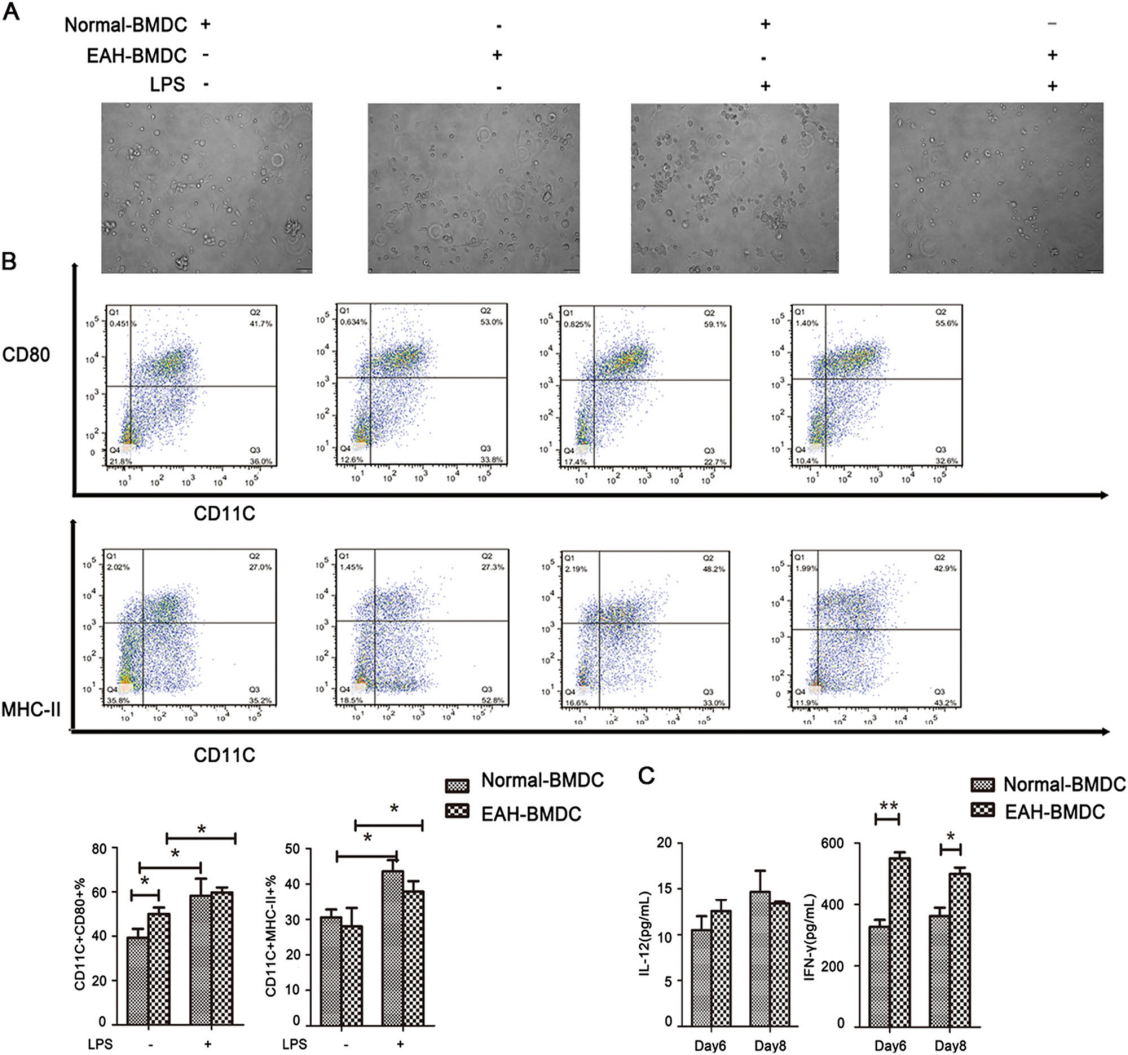
Pro-inflammatory function of BMDCs from EAH mice. **a** Morphology and phenotypic characteristics of BMDCs at day 8 treated with LPS. BMDCs were treated with or without LPS for 24 h. **b** FACS analysis of mature status when culture of BMDCs from normal and AIH mice before and after LPS challenged. **c** ELISA analysis of cytokines released of induced BMDCs on day 6 and day 8 from normal and AIH mice. *p < 0.05 and **p < 0.01.

After we determined that cytokine production and maturation were major functions of cDCs with great biological importance, we then investigated whether maturation marker expression and secreted concentrations of cytokines of induced BMDCs were different between EAH mice and normal controls. Induced im-BMDCs on day 8 from EAH and normal mice were stimulated with LPS. As shown in Fig. 3a, no obvious change in the morphology of BMDCs from normal or EAH mice was observed in the presence or absence of LPS. However, flow cytometry analysis revealed the pro-inflammatory function of BMDCs from EAH mice, as evidenced by higher expression levels of MHC-II and CD80 compared with control cells before and after LPS challenge (Fig. 3b). BMDCs from EAH mice produced significantly higher levels of IFN-γ in the supernatant compared with BMDCs from normal controls. We also measured the levels of IL-12 in cell culture supernatants between the two groups without finding significant differences (Fig. 3c).

### ConA trigger phenotypic characteristics of BMDCs by aberrant regulation of autophagy in vitro

The mannose receptor is expressed by human and murine DCs generated in vitro, human monocyte-derived DCs (moDCs) and mouse BMDCs. Studies have found that uptake of mannosylated ligands by moDCs leads to the delivery of antigen to MHC-II and enhanced presentation to T cells^20,21^. ConA treatment was then performed to further explore the underlying mechanism in vitro.

**Fig. 4:**
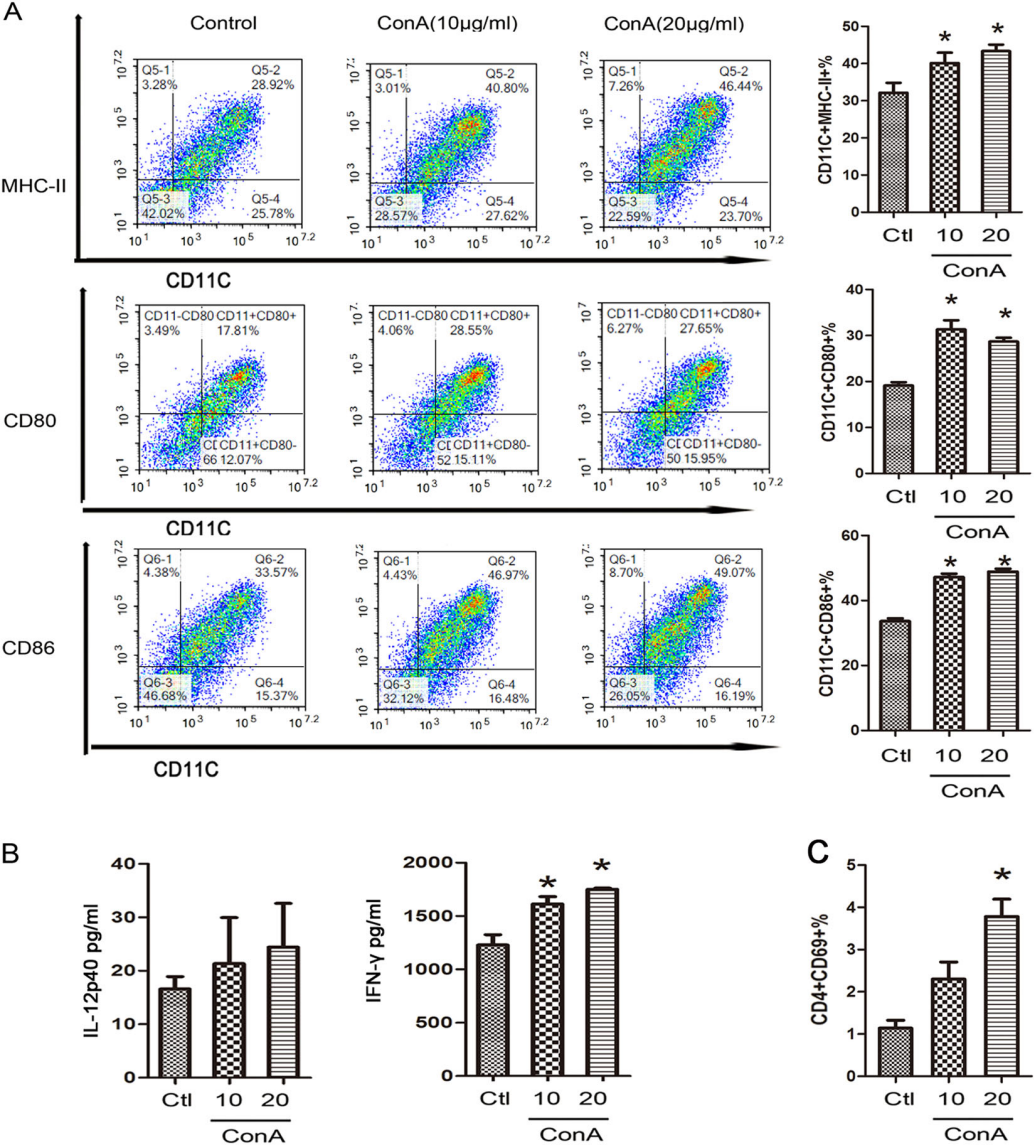
ConA trigger phenotypic characteristics of BMDCs in vitro. **a** Impact of ConA treatment on MHC-II and surface costimulatory molecules on BMDCs in each group. **b** ELISA analysis of cytokines released by BMDCs after ConA stimulation. **c** CD69 expression in T cells surface in each group. *p < 0.05 versus control.

Given the vital role of cDCs in Th1 cell differentiation and sensitization, the expression of MHC-II and costimulatory molecules on BMDCs was determined after ConA administration. As shown in Fig. 4a, the levels of MHC-II, CD80 and CD86 were significantly upregulated by ConA treatment. ConA treatment also caused a significant upregulation of IFN-γ in BMDCs in the presence of ConA. However, it did not affect the secretion of IL-12 (Fig. 4b). To determine whether ConA-induced mature BMDCs exhibited pro-inflammatory functions, we examined the effect of ConA-induced BMDCs on T-cell activation in vitro. The pro-inflammatory effects of BMDCs were determined when ConA-induced BMDCs were co-cultured with CD4+ T cells. BMDCs induced by ConA for 24 h exhibited a pro-inflammatory ability as evidenced by the higher positive percentage of CD4+ CD69+ T cells (Fig. 4c).

We then investigated the mechanisms involved in BMDCs maturation in EAH model, which is the recognized animal model of AIH. Accumulating evidence has shown that, in AIH patients and EAH model, the serum levels of LPS, TNF-α and IL-33 were markedly increased compared with that in healthy controls^22–24^. To reveal whether those elevated cytokines in AIH patients or EAH model could induce the maturation of BMDCs, im-BMDCs were stimulated in vitro with LPS, TNF-α and IL-33, respectively. As shown in Supplementary Fig. 4, LPS, TNF-α and IL-33 were demonstrated to greatly accelerate the maturation of BMDCs.

**Fig. 5:**
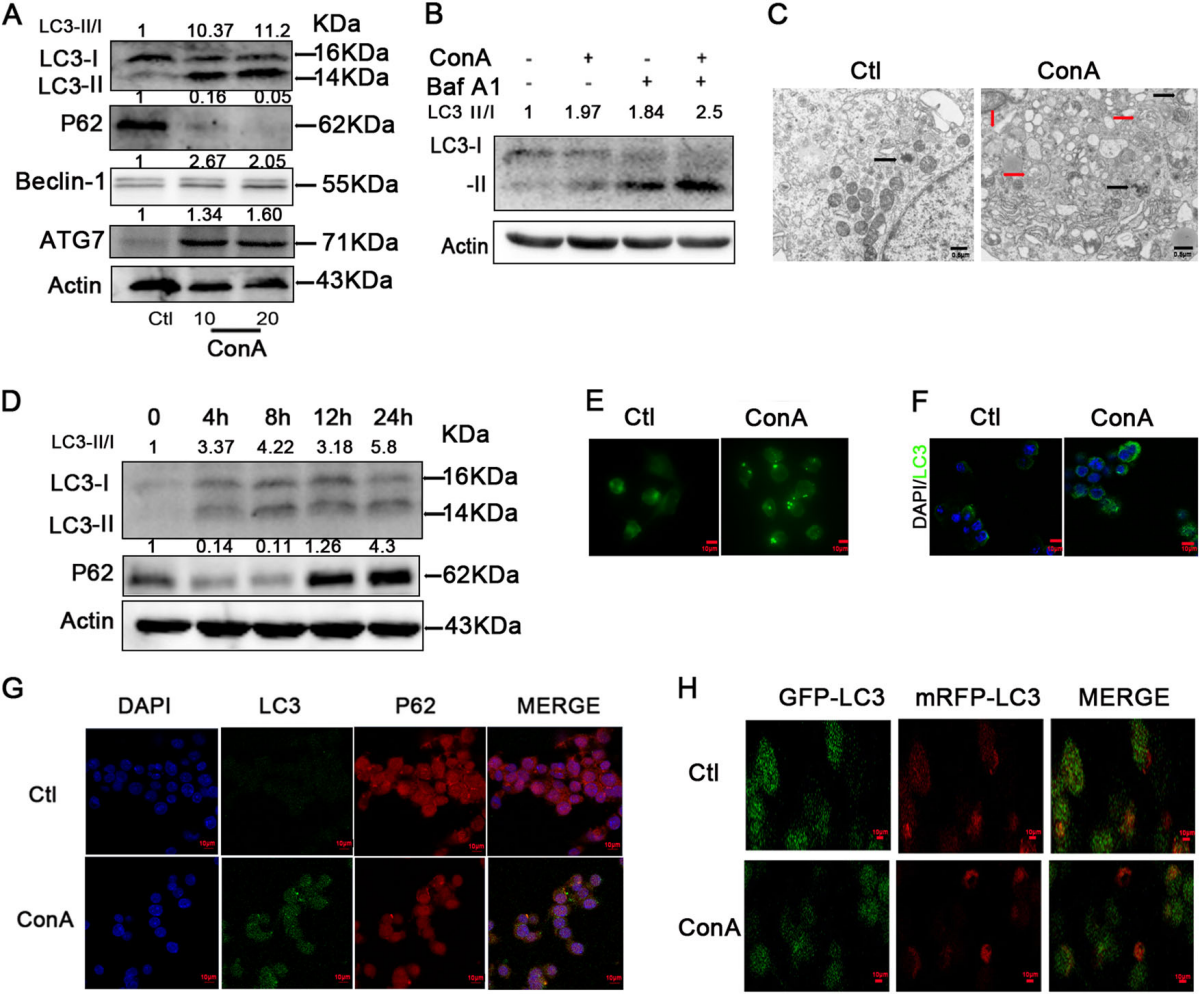
ConA stimulation induces autophagy in BMDCs and DC2.4 cell line. **a** Western blotting analysis of LC3-II,P62, Beclin-1 and ATG7 in BMDCs in each group; **b** Autophagic flux was calculated by dividing the levels of LC3-II in the presence of bafilomycin A1 by that without bafilomycin A1; **c** Transmission electron microscopy examination of autophagosomes and autolysosomes in the BMDCs. Autophagosomes were indicated by black arrows, and autolysosomes were indicated by red arrows; **d** Western blotting analysis of LC3-II and P62 in DC2.4 cell line in each group; **e** Acidic vacuoles in DC2.4 cells by ConA treatment, were stained with MDC and then observed under a fluorescence microscope; **f** LC3B-positive puncta in DC2.4 were examined for identifying autophagosome formation by fluorescence staining; **g** Immunofluorescence of LC3-II and p62 upon ConA treatment; **h** ConA-induced autophagy was evaluated by GFP-mRFP-LC3 plasmid transfection.

To investigate a link between ConA-induced BMDC maturation and autophagy, we measured the expression of the critical and classic autophagy-associated protein. After ConA treatment of BMDCs over concentration, we observed that the expression of robust LC3 conversion (i.e., cytosolic LC3I to autophagosome-bound lipidated LC3II) and decreased p62 levels in BMDCs, as well as the increased expression of ATG7 and Beclin-1 (Fig. 5a). Then we used 10 μg/mL ConA stimulation for 24 h in BMDCs in vitro for subsequent studies. As the fact that the increased autophagosomes may be due to either autophagy induction or inhibition of autophagic flux, bafilomycin-A, a specific inhibitor of vacuolar-type H + ATPase, which inhibits late stages of autophagy by preventing the fusion of autophagosome-lysosom, was used to explicit the effect of ConA on autophagy flux. As is shown in Fig. 5b, the LC3-II levels were further up-regulated in the presence of bafilomycin-A1 treatment, indicating that the upregulation of LC3-II was actually caused by activation of autophagy pathway, rather than blocking of autophagy flux. As shown in Fig. 5c, TEM images showed more autophagosomes and autolysosomes in the ConA-treated group than in the control group.

DC2.4 is a cell line derived from mouse bone marrow, and in the inactive form of the cells. DC2.4 was performed to have more controls for robust conclusions of autophagy flux in the process. First, exposure of cells to ConA over time induced robust LC3 conversion, while the P62 level was increased after 8 h (Fig. 5d). Hence, we used 10ug/mL ConA stimulation for 8 h in DC2.4 in vitro for further study. Then we used MDC, an acidotropic dye which tends to accumulate in late stage autophagosome like vacuoles to monitor the effect of ConA on autophagy. The results showed that significantly more MDC-labeled vacuoles accumulated in the cytoplasm of DC2.4 cells in the ConA-treated group than in the control group. Next, we further confirmed our results by examining the degree of autophagosome formation induced by ConA treatment of DC2.4 by immunofluorescent staining, and the expression analysis of LC3B and p62 conformed to the immunoblotting (Fig. 5e-g).

To confirm that the results of immunofluorescence and immunoblotting were indeed related to autophagy, the effect of ConA treatment on autophagy flux was further investigated using a tandem-tagged GFP-mRFP-LC3 plasmid. In our study, the results suggest that ConA could cause the activation of autophagy pathway, while not blocking the autophagosome-lysosome fusion in DC2.4 cell line (Fig. 5h).

### Effect of autophagy alteration on the phenotypic maturation of ConA‑induced BMDCs

Next, we aimed to determine whether the regulation of autophagy could affect the ConA-induced phenotypic maturation of BMDCs. The autophagy inhibitor 3-MA (3 mM) and bafilomycin A1, were used to exhibit the inhibitory effect.

**Fig. 6:**
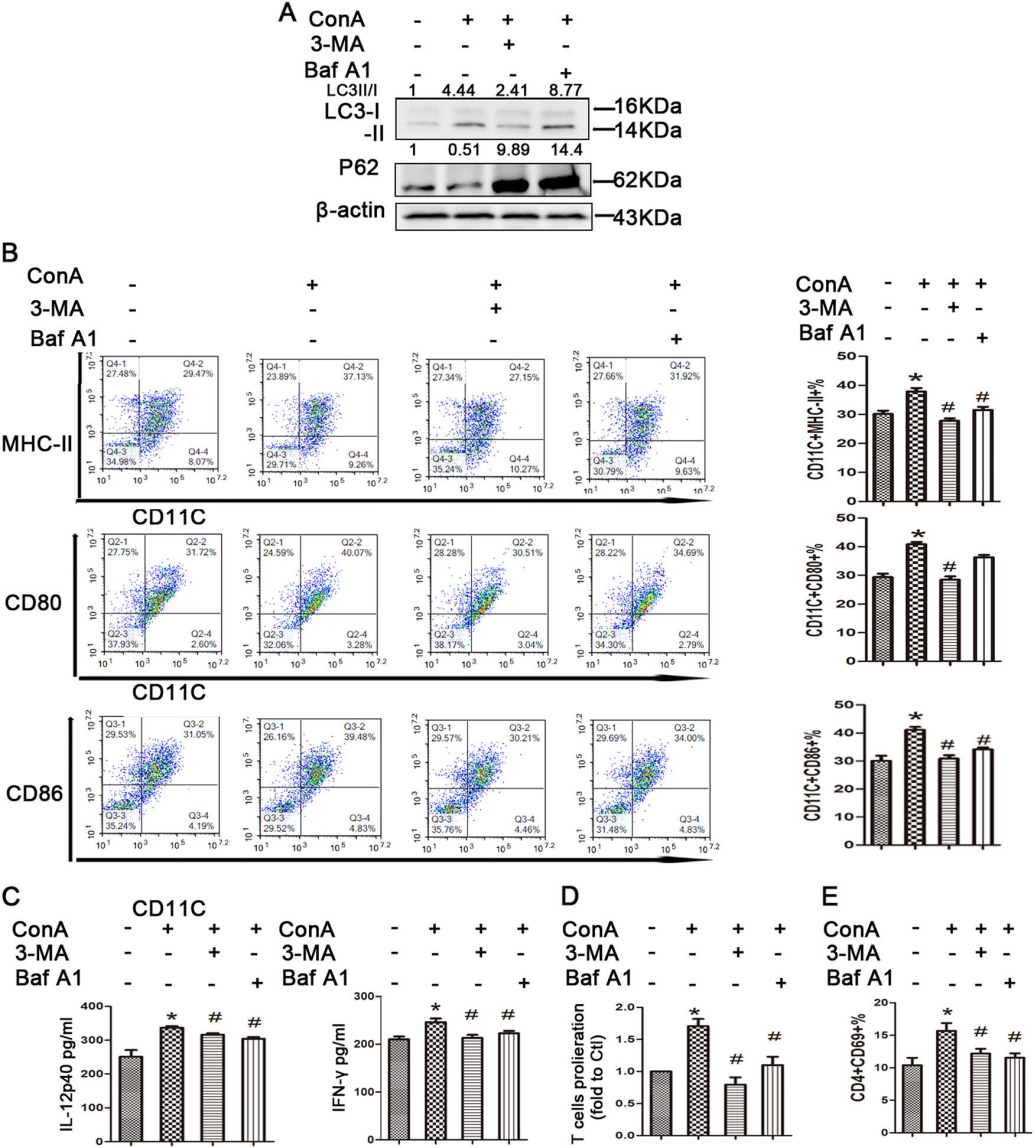
Effect of autophagy inhibitors on ConA‑induced BMDCs. **a** Western blotting analysis of LC3-II and P62 levels in BMDCs in each group; **b** impact of autophagy inhibitors treatment on MHC-II and surface costimulatory molecules on BMDCs in each group. **c** ELISA analysis of cytokines released by BMDCs after autophagy inhibitors stimulation. **d** BMDCs-induced proliferation of T cells and CD69 expression in T cells surface in each group. *p < 0.05 versus control; ^#^*p* < 0.05 versus ConA treatment.

We then treated ConA induced BMDCs with or without 3-MA (3 mM) 0.5 h, or bafilomycin-A1 1 h, prior to the harvest of cells, respectively. As shown in Fig. 6a, b, 3-MA or bafilomycin-A1, altered autophagy level in BMDCs, as evidenced by the expression of the LC3B-II/LC3B-I ratios in protein levels. Then, we evaluated the maturation markers CD11c, MHC-II, CD80 and CD86 using flow cytometry. The ConA-induced, autophagy inhibitor-treated BMDCs expressed significantly lower levels of MHC-II, CD80 and CD86 compared to ConA-induced BMDCs only (Fig. 6b). The secretion of IL-12 and IFN-γ in Con A-induced BMDCs was significantly reduced in the presence of both autophagy inhibitors (Fig. 6c). Next, BMDCs were incubated with ConA in the presence or absence of autophagy inhibitor for 24 h, and subsequently co-cultured with OVA-specific naive CD4+ T cells. After 3 d, CD4+ T-cell proliferation was measured by a CFSE dilution assay, and CD4+ T-cell activation was assessed by flow cytometry. We found that the addition of ConA drastically increased CD4+ T-cell proliferation and activation, while autophagy inhibitor treatment showed a decreased ability to induce T-cell proliferation and activation. (Fig. 6d, e) (Supplementary Fig. 6). These results suggest that autophagy plays a critical role in the maturation of BMDCs stimulated by ConA in vitro.

## Discussion

We report that abnormal maturation of cDCs in modulation of T-cell proliferation and function suggests pro-inflammatory cDCs role in progression with AIH. Furthermore, we also provided evidence that aberrant autophagy activity could be an important mechanism for DCs to promote the development of AIH. Furthermore, our studies revealed that inhibiting autophagy may be a promising therapeutic strategy to induce tDCs for the potential treatment of AIH.

Antigen presentation to CD4+ T helper cells is mediated by HLA-DR molecules. The quantitative expression of this molecule can affect the initiation of immune responses. Therefore, an increase in this molecule might lead to the overreaction of cDCs, which results in systematic immune imbalance. Quantitative and functionality alterations of human DC cells have been reported in many autoimmune diseases^25–29^, yet the role of cDC in AIH has been elusive. Using the fresh peripheral blood and liver section of AIH patients and healthy controls, we demonstrated that mature cDCs played a detrimental role in promoting AIH, and then, a mouse EAH model was used to validate the phenomenon. Variation in costimulatory molecules (CD80, CD86 and CD40) and MHC-II on DCs induces DC maturation and is considered a key event in T-cell activation. Our data demonstrate an increase in the frequency of mature cDCs in the periphery and liver tissues of AIH, which is paralleled by an increase in ALT. Yokomori H. et al. suggested that the presence of activated DCs in the hepatic sinusoids and central vein may be an important marker of AIH by electron microscopy founding^30^. Wang et al. demonstrated a detrimental role of cDCs in ConA-induced hepatitis by depletion of cDCs using either CD11c-diphtheria toxin receptor transgenic mice (DTR Tg) mice or anti-CD11c antibody with reduced the severity of liver injury significantly^31^. The matured of BMDCs by different stimulus such as TNF-α and IL-33, which were reported to be increased in AIH patients and EAH model, indicated that the mature of DCs may be related to the pathogenesis of AIH. We also analyzed that the percentage of mature BMDCs from EAH mice was increased compared with that of control mice. Even though further studies are required to prove these possibilities, our findings proposed that abnormal cDCs activation and recruitment may be associated with the pathogenesis of AIH.

We next addressed the question concerning how aberrant mature cDCs mediated EAH. The autophagy process is often used to eliminate damaged or unwanted organelles and remove intracellular microbial pathogens, which play a significant role in the pathogenesis of liver disease^32,33^. Role of autophagy in dendritic cells have been reported to be associated with the following mechanisms: (i) autophagic pathways could promote MHC-II antigen-presentation, that is, autophagy could be used to direct pathogens into autophagosomes and then the fusion with lysosomes,^28,34–36^ (ii) autophagy could down-regulate antigen presentation on MHC-I molecules during anti-viral CD8 + T cell responses,^37,38^ (iii) autophagy could accelerate cytokine production^39,40^. Most studies have found that autophagy enhancement was associated with BMDC maturation and antigen presentation after activation of Toll-like receptor (TLR) signaling pathways^41–43^, while other studies identified that new drug therapies could decrease DC maturation and function by enhanced autophagy^44,45^. In the current study, we provided evidence that enhanced autophagy activity is associated with the abnormal maturation of BMDCs during ConA administration, and inhibiting autophagy may induce the tDCs in vitro.

The efficacy of DCs depends on many variables, especially maturation status and efficient antigen presentation to naive T cells. Pro-inflammatory cytokines are required in the antigen presenting process of DCs and activating T cells. The switch of the DC to a pro-inflammatory state disrupts tolerance by activating and inducing differentiation of autoreactive T cells via T-cell receptor (TCR) ligation and cytokines such as IL-12, IL-6, IFN-γ^46,47^. IL-12 and IFN-γ are vital cytokines that increase naive CD4+ T-cell differentiation into Th1 cells. Our results revealed increased IL-12 and/or IFN-γ levels in the peripheral blood of AIH patients and in the culture supernatant of primary BMDCs. Autophagy inhibitors treatment in vitro decreased these cytokines in the culture supernatant of primary BMDCs, indicating that autophagy inhibitors impacts the differentiation of Th1 cells mediated by DC function.

Taken together, our results established that mature cDCs may contribute to the progression of AIH through excessive maturation. Aberrant autophagy flux plays an inhibitory role in the immunogenic maturation of DCs, and inhibition of autophagy flux on cDCs could be exploited as a new therapeutic approach for AIH.

## Supplementary information

Supplementary figure legend

Supplementary Figure 1

Supplementary Figure 2

Supplementary Figure 3

Supplementary Figure 4

Supplementary Figure 5

Supplementary Figure 6
