# Supplementary material for: Critical roles of conventional dendritic cells in autoimmune hepatitis via autophagy regulation
Source: Cell Death Dis. 2020 Jan 13;11(1):23. doi: 10.1038/s41419-019-2217-6 (PMC6957703; doi:10.1038/s41419-019-2217-6)
Supplement: Supplementary file 1 — Supplementary figure legend [file 41419_2019_2217_MOESM1_ESM.docx]

**Supplementary figure legend**

**Supplementary Figure 1**. Cell purity detection by FACS.

(A) The purity of imBMDCs by FACS. (B) The purity of CD4+T cells by FACS.

**Supplementary Figure 2**. Circulating activated T cells was increased in AIH patients.

Analysis of activated T cells in the peripheral blood of HCs and AIH patients. *p < 0.05 versus control.

**Supplementary Figure 3.** EAH model was successfully established.

(A)Serum ALT and AST levels. (B) Liver index and spleen index between the two groups. (C) Representative hematoxylin and eosin‑stained sections of the liver (original magnification, x200) (D) Representative analysis of activated T cells were analyzed by FACS for the presence of CD4+CD69+ cells in blood and liver. (E) Frequency of activated T cells were analyzed by FACS for the presence of CD4+CD69+ cells in blood and liver. (F) IHC of CD4+ in the hepatic tissues between the two groups. *p < 0.05 and ***p < 0.001 versus control.

**Supplementary Figure 4.** The matured of BMDCs by different stimulus. Im-BMDCs were stimulated *in vitro* with LPS, TNF-α and IL-33 for 24h, respectively, and surface marker expression was assessed by FACS. *p < 0.05 versus control.

**Supplementary Figure 5. The matured of DC 2.4 cell line by ConA.** DC2.4 was stimulated *in vitro* with ConA for 8h and surface marker expression was assessed by FACS. *p < 0.05 and **p<0.01 versus control

**Supplementary Figure 6**. Representative image of CD4 + T-cell proliferation pre-labeled by CFSE at 72h. T cells were pre-labeled with CFSE (37℃) for 15 minutes and then cultured with the BMDCs of different groups for 72h.
