# Supplementary figures and images for: Critical roles of conventional dendritic cells in autoimmune hepatitis via autophagy regulation
Source: Cell Death Dis. 2020 Jan 13;11(1):23. doi: 10.1038/s41419-019-2217-6 (PMC6957703; doi:10.1038/s41419-019-2217-6)

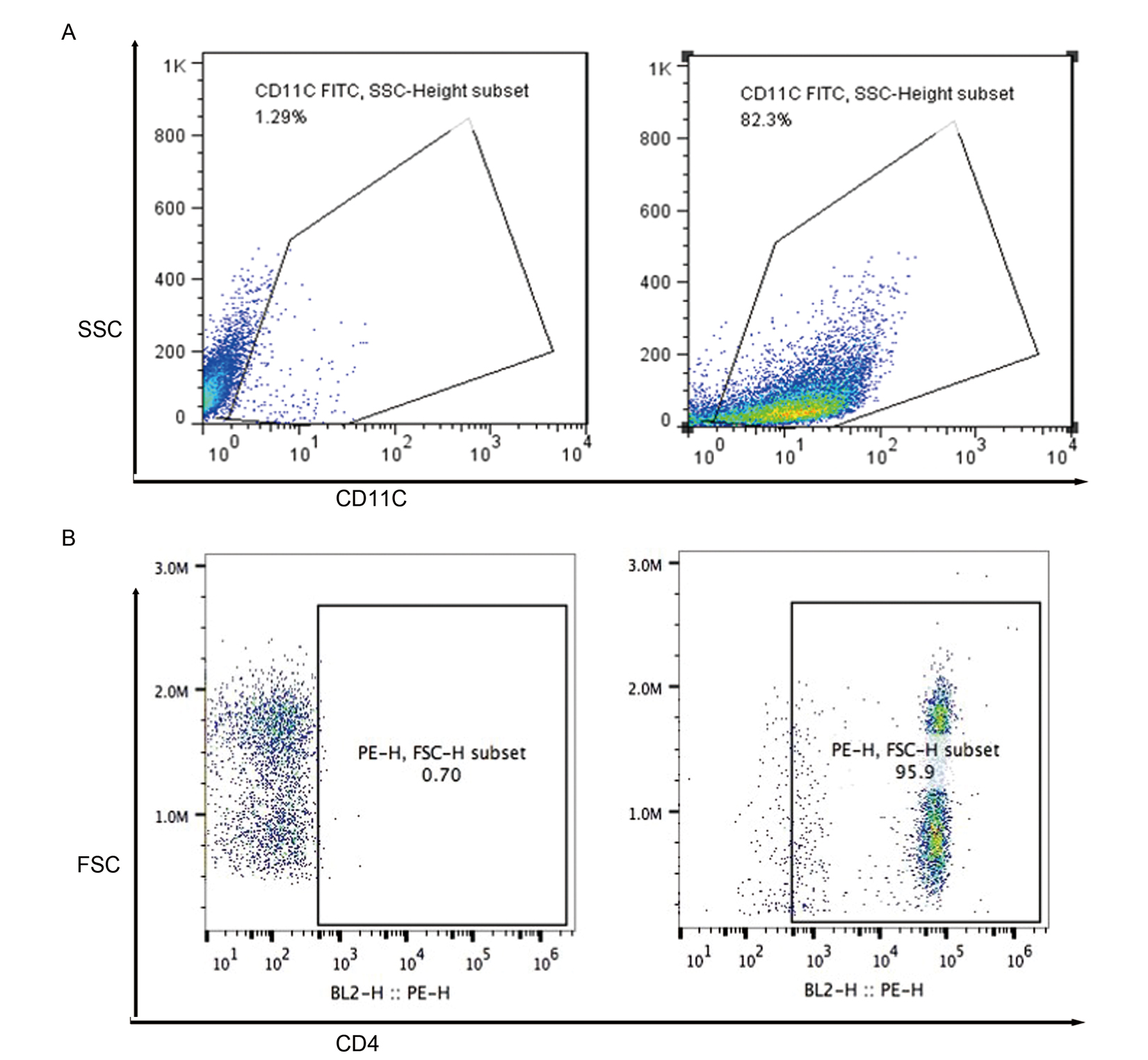

Supplement: Supplementary file 2 — Supplementary Figure 1 [file 41419_2019_2217_MOESM2_ESM.png]

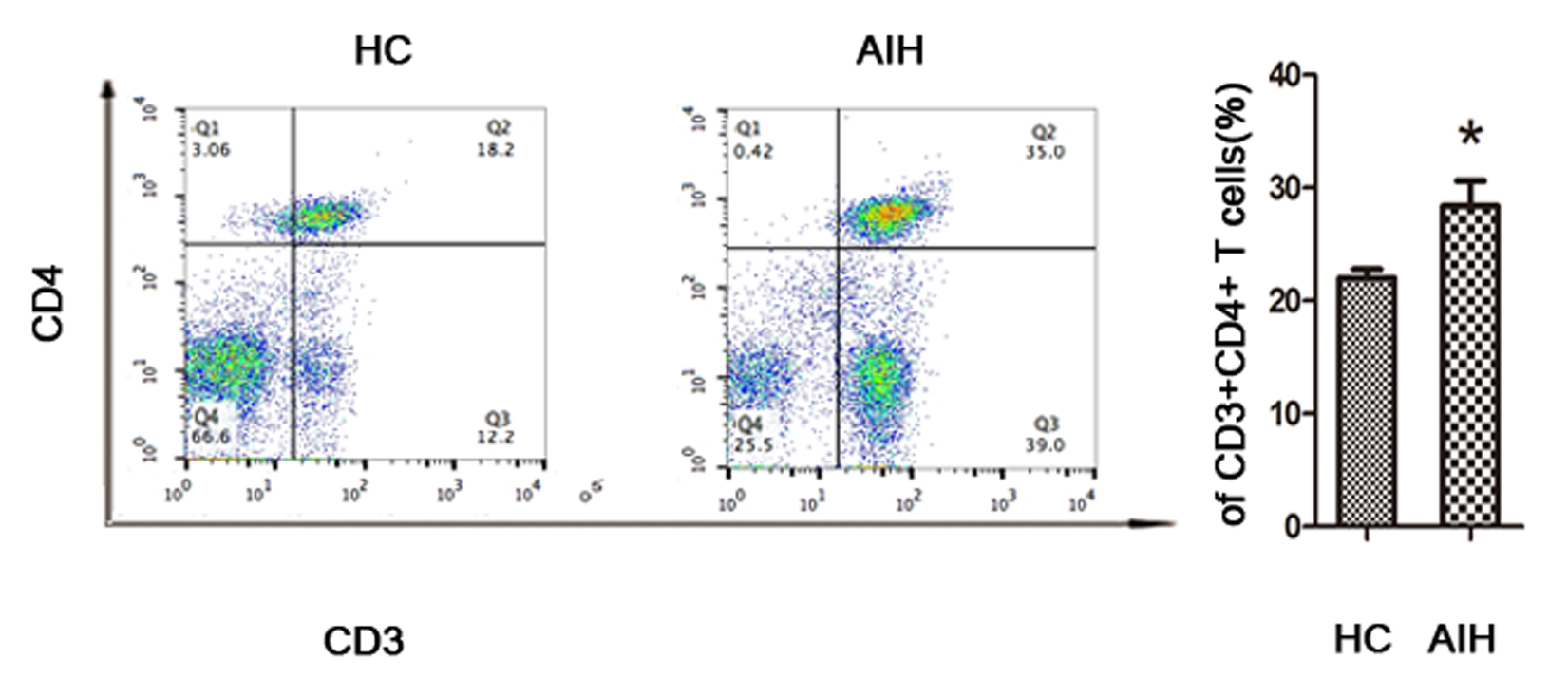

Supplement: Supplementary file 3 — Supplementary Figure 2 [file 41419_2019_2217_MOESM3_ESM.png]

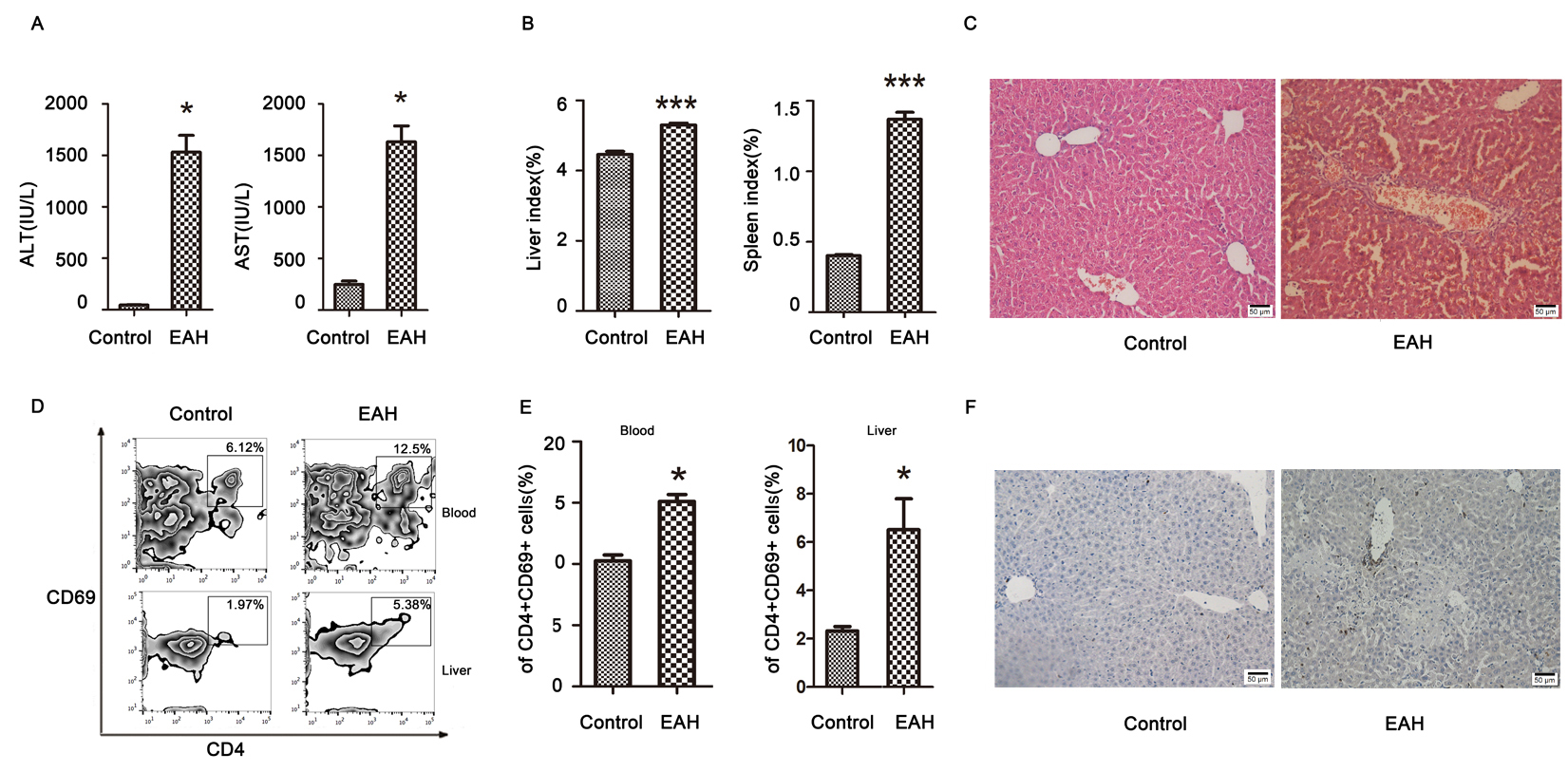

Supplement: Supplementary file 4 — Supplementary Figure 3 [file 41419_2019_2217_MOESM4_ESM.png]

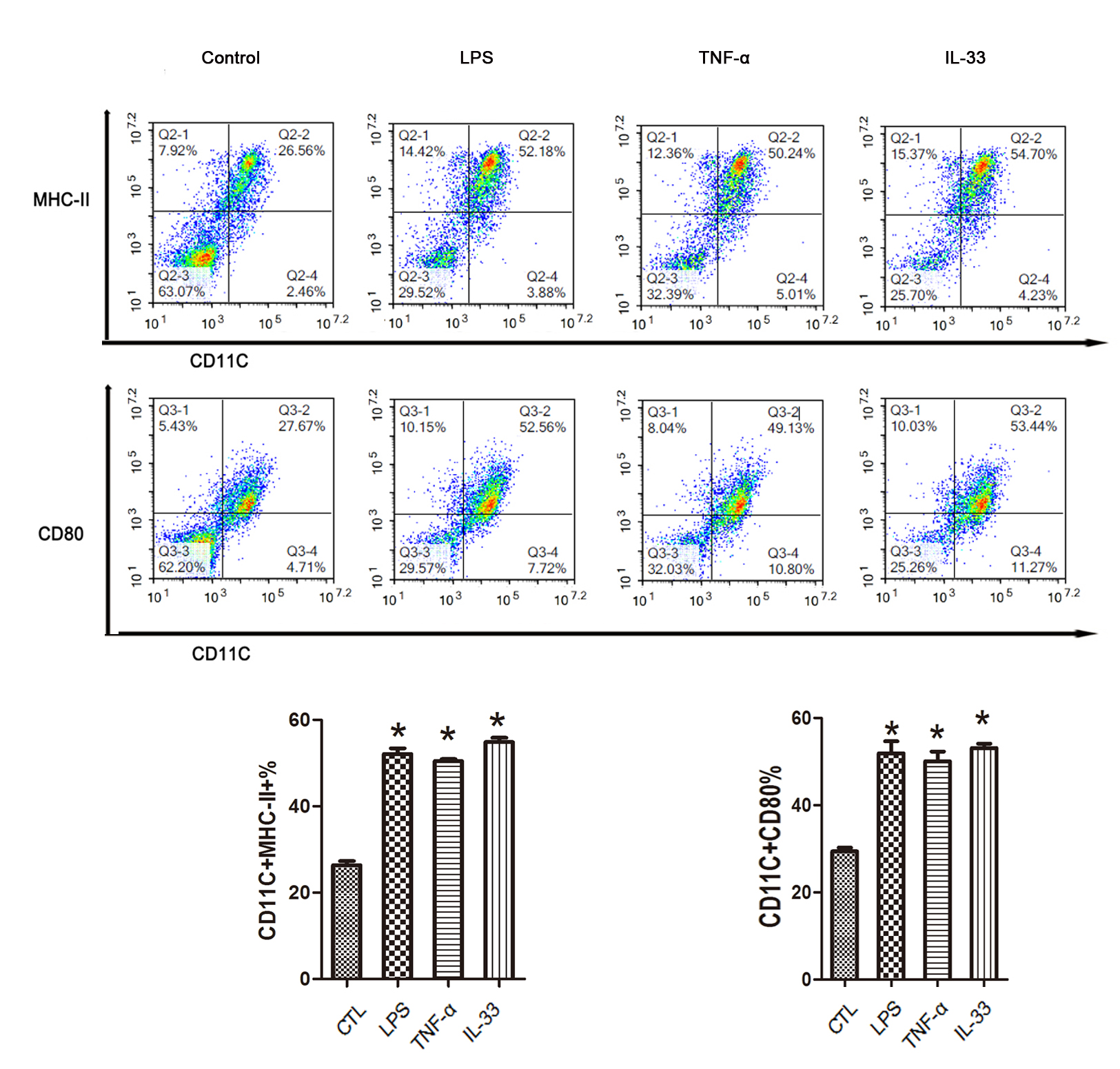

Supplement: Supplementary file 5 — Supplementary Figure 4 [file 41419_2019_2217_MOESM5_ESM.png]

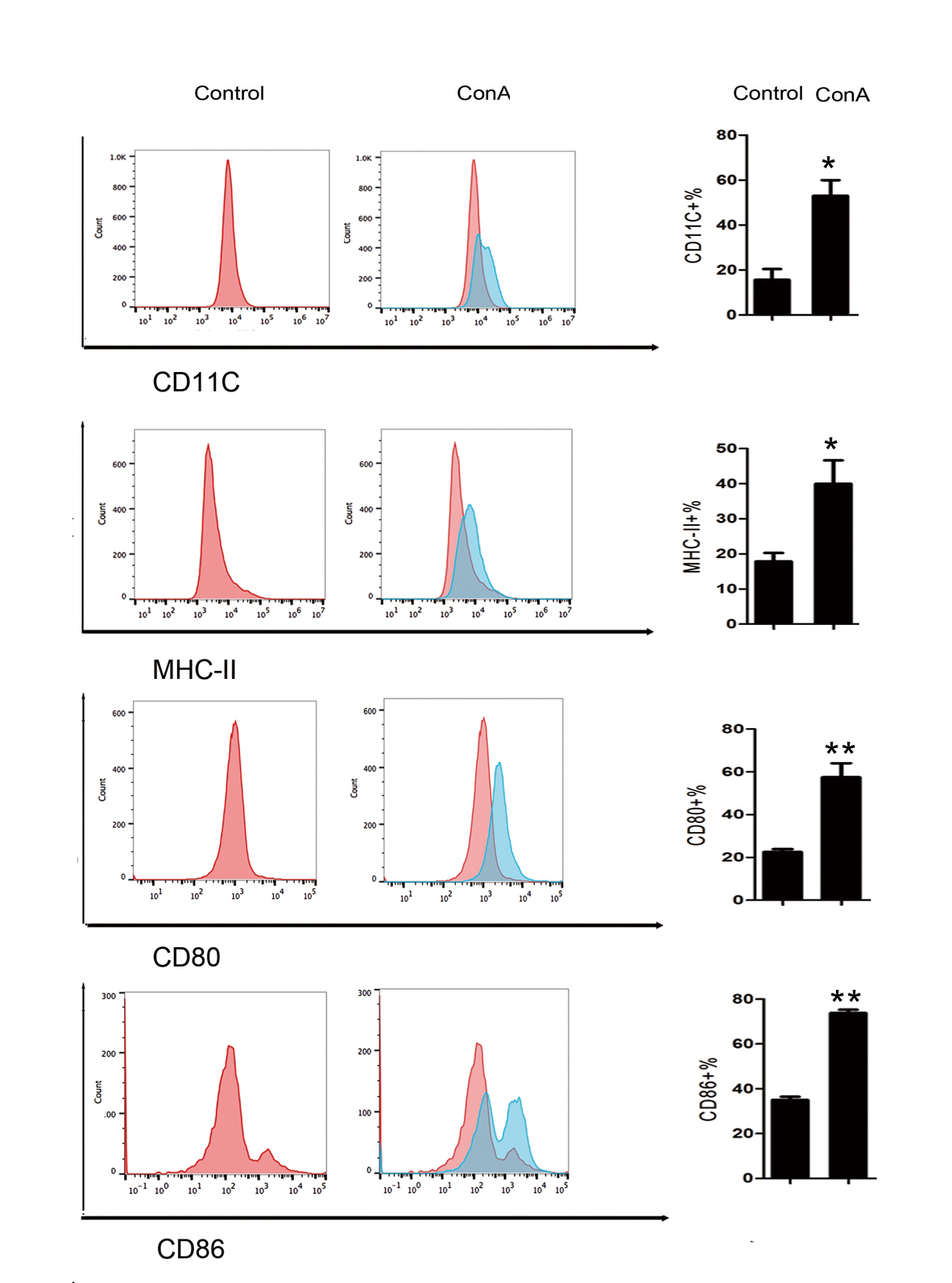

Supplement: Supplementary file 6 — Supplementary Figure 5 [file 41419_2019_2217_MOESM6_ESM.png]

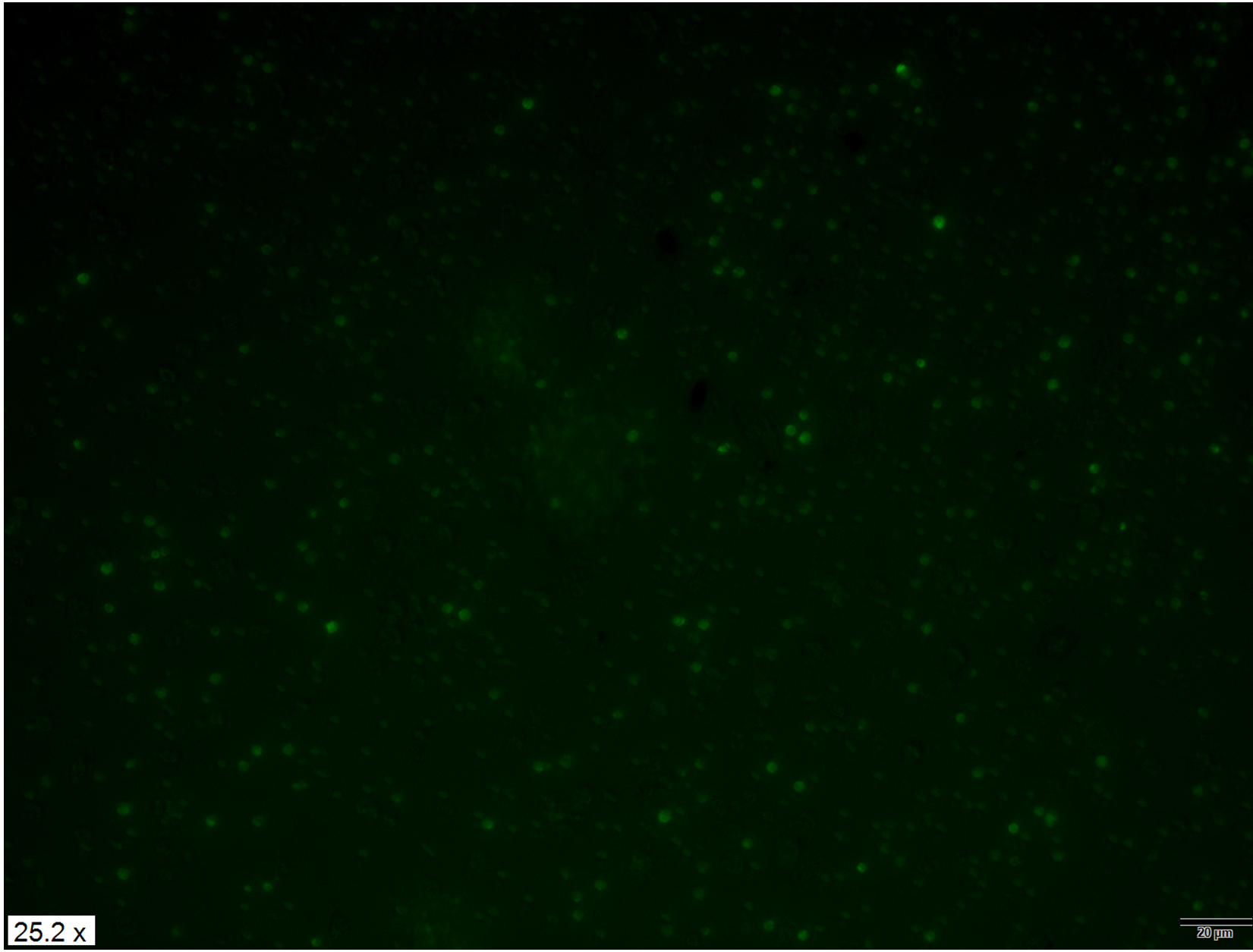

Supplement: Supplementary file 7 — Supplementary Figure 6 [file 41419_2019_2217_MOESM7_ESM.png]
